# The diagnosis of brain death

**DOI:** 10.4103/0972-5229.53108

**Published:** 2009

**Authors:** Ajay Kumar Goila, Mridula Pawar

**Affiliations:** **From:** Department of Anesthesia, Dr. Ram Manohar Lohia Hospital, New Delhi-110 001, India

**Keywords:** Apnoea test, brain stem function, brain stem reflexes, confounding and compatible conditions

## Abstract

Physicians, health care workers, members of the clergy, and laypeople throughout the world have accepted fully that a person is dead when his or her brain is dead. Although the widespread use of mechanical ventilators and other advanced critical care services have transformed the course of terminal neurologic disorders. Vital functions can now be maintained artificially for a long period of time after the brain has ceased to function. There is a need to diagnose brain death with utmost accuracy and urgency because of an increased awareness amongst the masses for an early diagnosis of brain death and the requirements of organ retrieval for transplantation. Physicians need not be, or consult with, a neurologist or neurosurgeon in order to determine brain death. The purpose of this review article is to provide health care providers in India with requirements for determining brain death, increase knowledge amongst health care practitioners about the clinical evaluation of brain death, and reduce the potential for variations in brain death determination policies and practices amongst facilities and practitioners. Process for brain death certification has been discussed under the following: 1. Identification of history or physical examination findings that provide a clear etiology of brain dysfunction. 2. Exclusion of any condition that might confound the subsequent examination of cortical or brain stem function. 3. Performance of a complete neurological examination including the standard apnea test and 10 minute apnea test. 4. Assessment of brainstem reflexes. 5. Clinical observations compatible with the diagnosis of brain death. 6. Responsibilities of physicians. 7. Notify next of kin. 8. Interval observation period. 9. Repeat clinical assessment of brain stem reflexes. 10. Confirmatory testing as indicated. 11. Certification and brain death documentation.

## Introduction

In the practice of critical care, ‘the care of a severely brain injured patient ’ is one of the toughest challenges for a critical care physician. Initial therapy provided for patients with severe brain injury or insult, is directed towards preservation and restoration of neuronal function. When this primary treatment is unsuccessful and the patient's condition evolves to brain death, the critical care physician has the responsibility to diagnose brain death with certainty and to offer the patient's family the opportunity to donate organs and / or tissues.

There is a clear difference between severe brain damage and brain death. The physician must understand this difference, as brain death means that life support is futile, and brain death is the principal prerequisite for the donation of organs for transplantation.

This review focuses on the clinical determination of brain death in adults and children, including the potential confounding factors, and provides an overview of valid confirmatory tests

## Evolution of the criteria for brain death

Historically death was defined by the presence of putrefaction or decapitation, failure to respond to painful stimuli, or the apparent loss of observable cardio respiratory action. The widespread use of mechanical ventilators that prevent respiratory arrest has transformed the course of terminal neurologic disorders. Vital functions can now be maintained artificially after the brain has ceased to function. In 1968, an ad hoc committee at Harvard Medical School reexamined the definition of brain death and defined irreversible coma, or brain death, as unresponsiveness and lack of receptivity, the absence of movement and breathing, the absence of brain-stem reflexes, and coma whose cause has been identified.

## Definition

Brain death is defined as the irreversible loss of all functions of the brain, including the brainstem. The three essential findings in brain death are coma, absence of brainstem reflexes, and apnoea. An evaluation for brain death should be considered in patients who have suffered a massive, irreversible brain injury of identifiable cause. A patient determined to be brain dead is legally and clinically dead.

The diagnosis of brain death is primarily clinical. No other tests are required if the full clinical examination, including each of two assessments of brain stem reflexes and a single apnoea test, are conclusively performed.

## Determination of brain death

### The process for brain death certification includes

1. Identification of history or physical examination findings that provide a clear etiology of brain dysfunction.
  The determination of brain death requires the identification of the proximate cause and irreversibility of coma. Severe head injury, hypertensive intracerebral hemorrhage, aneurysmal subarachnoid hemorrhage, hypoxic-ischemic brain insults and fulminant hepatic failure are potential causes of irreversible loss of brain function.
  The evaluation of a potentially irreversible coma should include, as may be appropriate to the particular case; clinical or neuro-imaging evidence of an acute CNS catastrophe that is compatible with the clinical diagnosis of brain death;
2. Exclusion of any condition that might confound the subsequent examination of cortical or brain stem function. The conditions that may confound clinical diagnosis of brain death are:
  - Shock/ hypotension
  - Hypothermia -temperature < 32°C
  - Drugs known to alter neurologic, neuromuscular function and electroencephalographic testing, like anaesthetic agents, neuroparalytic drugs, methaqualone, barbiturates, benzodiazepines, high dose bretylium, amitryptiline, meprobamate, trichloroethylene, alcohols.
  - Brain stem encephalitis.
  - Guillain- Barre' syndrome.
  - Encephlopathy associated with hepatic failure, uraemia and hyperosmolar coma
  - Severe hypophosphatemia.
3. Performance of a complete neurological examination. Components of a complete neurological examination are:
  - Examination of the patient-absence of spontaneous movement, decerebrate or decorticate posturing, seizures, shivering, response to verbal stimuli, and response to noxious stimuli administered through a cranial nerve path way.
    During the examination spinal reflexes may be present.
  - Absent pupillary reflex to direct and consensual light; pupils need not be equal or dilated. The pupillary reflex may be selectively altered by eye trauma, cataracts, high dose dopamine, glutethamide, scopolamine, atropine, bretilium or monoamine oxidase inhibitors.
  - Absent corneal, oculocephalic, cough and gag reflexes. The corneal reflex may be altered as a result of facial weakness.
  - Absent oculovestibular reflex when tested with 20 to 50 ml. Of ice water irrigated into an external auditory canal clear of cerumen, and after elevating the patients head 30'. Labyrinthine injury or disease, anticholinergics, anticonvulsants, tricyclic antidepressants, and some sedatives may alter response.
  - Failure of the heart rate to increase by more than 5 beats per minute after 1- 2 mg. of atropine intravenously. This indicates absent function of the vagus nerve and nuclei.
  - Absent respiratory efforts in the presence of hypercarbia.
    Generally, the apnoea test is performed after the second examination of brainstem reflexes.
    The apnoea test need only be performed once when its results are conclusive. Before performing the apnoea test, the physician must determine that the patient meets the following conditions:
    - Core temperature ≥ 36.5°C or 97.7°F
    - Euvolemia. *Option:* positive fluid balance in the previous 6 hours
    - Normal PCO_2_. *Option:* arterial PCO_2_ ≥ 40 mm Hg
    - Normal PO_2_. *Option:* pre-oxygenation to arterial PO_2_ ≥ 200 mm Hg

After determining that the patient meets the above prerequisites, the physician should conduct the apnoea test as follows:

1. Connect a pulse oximeter and disconnect the ventilator.
2. Deliver 100% O_2_, 6 l/min, into the trachea. *Option:* place a cannula at the level of the carina.
3. Look closely for any respiratory movements (abdominal or chest excursions that produce adequate tidal volumes).
4. Measure arterial PO_2_, PCO_2_, and pH after approximately 8 minutes and reconnect the ventilator.
5. If respiratory movements are absent and arterial PCO_2_ is ≥ 60 mm Hg (*option:* 20 mm Hg increase in PCO_2_ over a baseline normal PCO_2_), the apnoea test result is positive (i.e. it supports the diagnosis of brain death).
6. If respiratory movements are observed, the apnoea test result is negative (i.e. it does not support the clinical diagnosis of brain death).
7. Connect the ventilator, if during testing
  - the systolic blood pressure becomes < 90 mm Hg (or below age appropriate thresholds in children less than 18 years of age)
  - or the pulse oximeter indicates significant oxygen desaturation,
  - or cardiac arrhythmias develop;

Immediately draw an arterial blood sample and analyze arterial blood gas.

- If PCO_2_ is ≥ 60 mm Hg or PCO_2_ increase is ≥ 20 mm Hg over baseline normal PCO_2_, the apnoea test result is positive (it supports the clinical diagnosis of brain death).
- if PCO_2_ is < 60 mm Hg and PCO_2_ increase is < 20 mm Hg over baseline normal PCO_2_, the result is indeterminate and a confirmatory test can be considered.
- When appropriate a 10 min. apnoea test can be performed after preoxygenation for 10 minutes with an Fi0_2_ of 1.0 and normalization of patients PaCO_2_ to 40 mmHG.

### Assessment of brainstem reflexes

1. Pupils- no response to bright light Size: midposition (4 mm) to dilated (9 mm) (absent light reflex - cranial nerve II and III)
2. Ocular movement- cranial nerve VIII, III and VI
  - No oculocephalic reflex (testing only when no fracture or instability of the cervical spine or skull base is apparent)
  - No deviation of the eyes to irrigation in each ear with 50 ml of cold water (tympanic membranes intact; allow 1 minute after injection and at least 5 minutes between testing on each side)
3. Facial sensation and facial motor response
  - No corneal reflex (cranial nerve V and VII)
  - No jaw reflex (cranial nerve IX)
  - No grimacing to deep pressure on nail bed, supraorbital ridge, or temporo-mandibular joint (afferent V and efferent VII)
4. Pharyngeal and tracheal reflexes (cranial nerve IX and X)
  - No response after stimulation of the posterior pharynx
  - No cough response to tracheobronchial suctioning

### Clinical observations compatible with the diagnosis of brain death:

The following manifestations are occasionally seen and should not be misinterpreted as evidence for brainstem function:

- spontaneous movements of limbs other than pathologic flexion or extension response
- respiratory-like movements (shoulder elevation and adduction, back arching, intercostal expansion without significant tidal volumes)
- sweating, flushing, tachycardia
- normal blood pressure without pharmacologic support or sudden increases in blood pressure
- absence of diabetes insipidus
- deep tendon reflexes; superficial abdominal reflexes; triple flexion response
- Babinski reflex

## Responsibilities of Physicians Determining Brain Death

The diagnosis of brain death is primarily clinical. No other tests are required if the full clinical examination, including each of two assessments of brain stem reflexes and a single apnoea test, is conclusively performed. In the absence of either complete clinical findings consistent with brain death, or confirmatory tests demonstrating brain death, brain death cannot be diagnosed and certified. These guidelines apply to patients one year of age or older.

### Notify Next of Kin

The facility must make diligent efforts to notify the person closest to the patient that the process for determining brain death is underway. Consent need not be obtained but requests for reasonable accommodation based on religious or moral objections should be noted and referred to appropriate hospital staff. Where family members object to invasive confirmatory tests, physicians should rely on the guidance of hospital counsel and the ethics committee.

### Interval Observation Period

After the first clinical exam, the patient should be observed for a defined period of time for clinical manifestations that are inconsistent with the diagnosis of brain death. Most experts agree that a 6 hour observation period is sufficient and reasonable in adults and children over the age of 1 year. Longer intervals are advisable in young children.

### Repeat Clinical Assessment of Brain Stem Reflexes

The examination as described above should be repeated in full and documented. When clinical circumstances prohibit completion of any steps in the clinical examination, these should be documented.

### Confirmatory Testing as Indicated

When the full clinical examination, including both assessments of brain stem reflexes and the apnoea test, is conclusively performed, no additional testing is required to determine brain death.

In some patients, skull or cervical injuries, cardiovascular instability, or other factors may make it impossible to complete parts of the assessment safely. In such circumstances, a confirmatory test verifying brain death is necessary. These tests may also be used to reassure family members and medical staff.

Any of the suggested tests may produce similar results in patients with catastrophic brain damage who do not fulfill the clinical criteria of brain death. The confirmatory tests are.

- Angiography (conventional, computerized tomographic, magnetic resonance, and radionuclide): Brain death confirmed by demonstrating the absence of intracerebral filling at the level of the carotid bifurcation or Circle of Willis. The external carotid circulation is patent, and filling of the superior sagittal sinus may be delayed.
- Radionuclide angiography (CRAG) does not adequately image vasculature of the posterior fossa.
- MRI angiography can be quite challenging in an ICU patient because of magnet incompatibility with lines, ventilator tubing and other hardware.
- Cerebral arteriography: This test is often difficult to perform in a critically ill, unstable patient.
- Electroencephalography: Brain death confirmed by documenting the absence of electrical activity during at least 30 minutes of recording that adheres to the minimal technical criteria for EEG recording in suspected brain death as adopted by the American Electroencephalographic Society, including 16-channel EEG instruments. The ICU setting may result in false readings due to electronic background noise creating innumerable artifacts.
- Nuclear brain scanning: Brain death confirmed by absence of uptake of isotope in brain parenchyma and/or vasculature, depending on isotope and technique used. (“hollow skull phenomenon”).
- Somatosensory evoked potentials: Brain death confirmed by bilateral absence of N20-P22 response with median nerve stimulation. The recordings should adhere to the minimal technical criteria for somatosensory evoked potential recording in suspected brain death as adopted by the American Electroencephalographic Society.
- Transcranial doppler ultrasonography: Brain death confirmed by small systolic peaks in early systole without diastolic flow, or reverberating flow, indicating very high vascular resistance associated with greatly increased intracranial pressure.
- Since as many as 10% of patients may not have temporal insonation windows because of skull thickness, the initial absence of Doppler signals cannot be interpreted as consistent with brain death.

## Certification of Brain Death

Brain death can be certified by a single physician privileged to make brain death determinations. However, before a patient can become an organ donor, New York State law requires that the time of brain death must be certified by the physician who attends the donor at his death and one other physician, neither of whom shall participate in the process of transplantation. This requirement ensures that all evaluations meet accepted medical standards, and that all participants can have confidence that brain death determination has not been influenced by extraneous factors, including the needs of potential organ recipients.

When two physicians are required to certify the time of death, i.e., when organ donation is planned, both physicians should affirm that the clinical evaluation meets accepted medical standards, and that the data fully support the determination of brain death. Generally, both physicians should observe the patient, review the medical record, and note whether any additional information is required to make a definitive determination. Neither physician should certify brain death unless all aspects of the determination have been completed.

*Medical Record Documentation*: All phases of the determination of brain death should be clearly documented in the medical record; The medical record must indicate:

- etiology and irreversibility of coma / unresponsiveness
- absence of motor response to pain
- absence of brainstem reflexes during two separate examinations separated by at least 6 hours
- absence of respiration with pCO_2_ ≥ 60 mm hg
- justification for, and result of, confirmatory tests if used

## Withdraw cardio-respiratory support in accordance with hospital policies, including those for organ donation

When a patient is certified as brain dead and the ventilator is to be disconnected, the family should be treated with sensitivity and respect. If family members wish, they may be offered the opportunity to attend while the ventilator is disconnected. However, family members should be prepared for the possibly disturbing clinical activity that they may witness when organ donation is contemplated, ventilatory support will conclude in the operating room and family attendance is not appropriate.

## Appendix:

Determination of Brain Death in Children Less Than One Year of Age

1. General Policy Statement.
  The brains of infants and young children have increased resistance to damage and may recover substantial functions even after exhibiting unresponsiveness on neurological examination for longer periods as compared to adults. When applying neurological criteria to determine death in children younger than one year, longer observation periods are required.
2. The patient must not be significantly hypothermic or hypotensive for age.
3. Observation Periods According to Age.

The recommended observation period depends on the age of the patient and the laboratory tests utilized. It is assumed that the child was born at full term Between the ages of 2 months and 1 year, an interval of at least 24 hours should be used. Between the ages of 7 days and 2 months, the minimum interval should be 48 hours.

1. Reliable criteria have not been established for the determination of brain death in children less than 7 days old.
2. Seven days to two months: Two examinations and electroencephalograms (EEGs) should be separated by at least 48 hours.
3. Two months to one year: Two examinations and EEGs should be separated by at least 24 hours. A repeat examination and EEG are not necessary if a concomitant radionuclide (CRAG) or other angiographic study demonstrates no visualization of cerebral arteries.
